# In-Line Mach Zehnder Interferometer Based on Ytterbium Doped Fiber with Up-Taper Structure in Fiber Ring Laser and Its Application in Sensing

**DOI:** 10.3390/s22239196

**Published:** 2022-11-26

**Authors:** Weihao Lin, Yuhui Liu, Perry Ping Shum, Liyang Shao

**Affiliations:** Department of Electrical and Electronic Engineering, Southern University of Science and Technology, Shenzhen 518055, China

**Keywords:** MZI, fiber ring laser, temperature sensor

## Abstract

We report an ytterbium (Yb) doped fiber Mach Zehnder interferometer (MZI) based on the up-taper fiber structure in a fiber ring laser (FRL) cavity. Different from the traditional FRL sensing system, in which additional filters are required, the designed structure simultaneously acts as a filter, sensor and gain medium. Furthermore, thanks to the high thermal–optical coefficient of Yb doped fiber, the temperature sensitivity of 0.261 nm/°C can be achieved in the range of 10–50 °C. In addition, benefiting from the unique characteristics of the laser system itself, the designed structure has a narrower linewidth (−0.2 nm) and a higher signal-to-noise ratio (SNR) (−40 dB) than the sensor system based on a broadband light source (BBS). Meanwhile, the refractive index (RI) response and stability of the system are measured. The RI sensitivity is up to 151 nm/RIU, and the wavelength fluctuation range within two hours is less than 0.2 nm. Therefore, the designed structure is expected to play a significant role in human life safety monitoring, aircraft engine temperature monitoring, etc.

## 1. Introduction

In recent years, optical fiber sensing technology has received extensive attention [1,2,3,4] because of its small size, light weight, low price and high sensitivity [5,6,7]. Its application range includes electromagnetic field detection [8,9,10,11], tumor marker measurement [12,13,14], and temperature or RI calibration [15,16,17,18,19,20], etc. Among them, optical fiber temperature and RI sensors have been widely reported because of immunity to electromagnetic interference and multiplexing capabilities [21,22,23,24]. However, the traditional optical fiber sensor uses a BBS as the light source [25,26,27], resulting in poor spectral quality and generate many burrs, which affects the accuracy and stability of the measurement.

An FRL cavity is gradually regarded as a substitute for the BBS because of its high SNR and narrow linewidth [28,29,30]. The interference structure is usually designed in the cavity as the filter unit. When the disturbance occurs in an external environment, the central wavelength of the system will shift. Liu et al. designed an RI sensor based on coreless fiber [31] and systematically studied the effects of wavelength and pulse width on the sensor property. The results demonstrate that the function of the sensing structure intensely depends on the interrogation wavelength, and the narrower pulse width helps to avoid the interference of relaxation oscillation. The detection sensitivity of −3271 µs/RIU is obtained. However, the designed structure requires photoelectric detectors and oscilloscopes to perform photoelectric conversion, which reduces the system efficiency. Mateusz et al. realized the simultaneous measurement of humidity and temperature by using a cascaded fiber Bragg grating (FBG) [32]. However, due to the inherent properties of the FBG, the detection sensitivity obtained is particularly low. In 2016, Zhao et al. made pioneering use of an up-taper MZI to measure temperature and RI in an FRL system, greatly improving the detection limit and SNR [33].

Here, we propose an MZI based on the up-taper structure of Yb doped fiber. In the FRL system, the cladding mode is excited by the first sphere and then recoupled in the second sphere after transmission to obtain interference. It benefits from the strong thermal–optical coefficient of rare earth optical fiber, and a temperature sensitivity of 0.261 nm/°C in the range of 10–50 °C was achieved. Furthermore, the RI response characteristics of the designed MZI was studied. It was found that the RI monitoring with sensitivity up to 144 pm/RIU could be realized. At the same time, the variation range of wavelength and intensity within 2 h is less than 0.2 nm and 0.5 dB, respectively, which verifies the practicability of the sensor. Benefiting from the inherent properties of the FRL, the linewidth of the designed structure is less than 0.2 nm, and the SNR is up to 40 dB. The sensor structure designed is expected to play a potential role in life health monitoring and stable operation of spacecraft.

## 2. Sensor Setup and Principle

**Figure 1:**
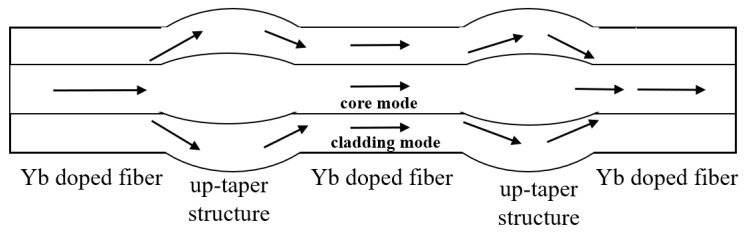
Schematic diagram of up-taper structure on Yb doped fiber.

The designed interferometer sensor structure is shown in Figure 1. MZI is composed of two cascaded Yb doped fiber up-taper structures. The output light is transmitted to the first spherical structure through the first section of Yb doped fiber. Firstly, the optical energy is transmitted in the fiber core in the modality of core mode. When the light transmission gets past the first sphere, the cladding mode is excited because of the mismatch of core diameter; when the core mode and cladding mode reach the second sphere, some higher-order cladding modes will recouple and then propagate along the fiber core. Since different modes have different phases, the interference between modes will occur.

The light intensity expression of the output interference spectrum can be expressed as:

(1) $$\;I=I_{core}+I_{clad}^{n}+2\sqrt{I_{core}+I_{clad}^{n}}cos\Delta \varphi \;$$

in which $I_{core}$ is the light intensity of core mode and $I_{clad}^{n}\;$is the light intensity of higher-order cladding mode. Because of diverse propagation constants among different modes, after the same transmission distance, there will exist phase difference between different modes. The higher-order cladding modes excited in the up-taper-structure-based MZI partake in the interference. The cladding modes of different orders are related to different effective RI, and the phase difference Δφ between the core mode and the n-step cladding mode can be expressed as:

(2) $$\Delta \varphi =\frac{2\pi \Delta n_{eff}L}{\lambda }=\frac{2\pi }{\lambda }\left(n_{eff}^{core}-n_{eff}^{cl,n}\right)L$$

where λ is the output light wavelength, $n_{eff}^{core}$ is the effective RI of core mode, $n_{eff}^{cl,n}\;$is the effective RI of different high order cladding mode, and L is the effective length of interference arm. The free spectral range of the interference spectrum can be expressed as:

(3) $$\Delta \lambda =\frac{\lambda^{2}}{\Delta n_{eff}^{c}L}$$

When the external temperature and refractive index change, the interference spectrum will shift. The expressions can be expressed as:

(4) $$\Delta \lambda \approx 2\lambda \left[\frac{1}{\Delta n_{eff}}\delta +k\right]\Delta T$$

(5) $$\Delta \lambda \approx \frac{-\lambda }{\Delta n_{eff}}\frac{\partial n_{eff}^{cl,n}}{\partial n_{RI}}$$

in which δ and k are the thermal–optical coefficient and thermal-expansion coefficient of the optical fiber, respectively. Since Yb doped fiber has higher thermal–optical coefficient than erbium doped fiber and single-mode fiber, higher temperature sensitivity can be achieved. $n_{RI}$ is the external RI value and $\Delta n_{eff}$ is the difference between cladding modes and core mode.

**Figure 2:**
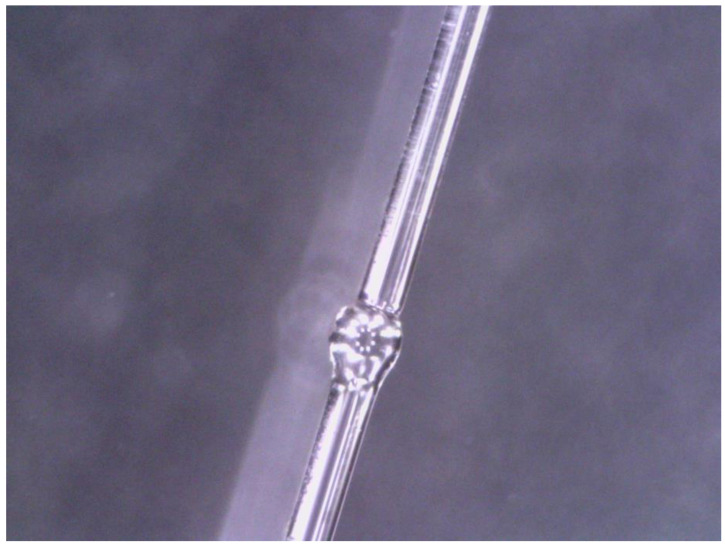
Micrograph of up-taper structure on Yb doped fiber.

The microstructure of the up-taper shape is shown in Figure 2. The structure can be easily fabricated by an optical fiber fusion splicer (Fujikura 80C, Japan). Firstly, we used the fiber cutter to smooth the end face of Yb doped fiber. Then, one end of the optical fiber was placed in the fusion splicer, discharging twice using parameters of +100 bit discharge power and 15,000 ms discharge time to form a smooth 325 μm spherical structure. The above operation was repeated to make another sphere. Finally, the two spheres were welded by a 1.5 cm Yb doped fiber to form the required structure after adjusting the welding mode to single mode welding mode. 

**Figure 3:**
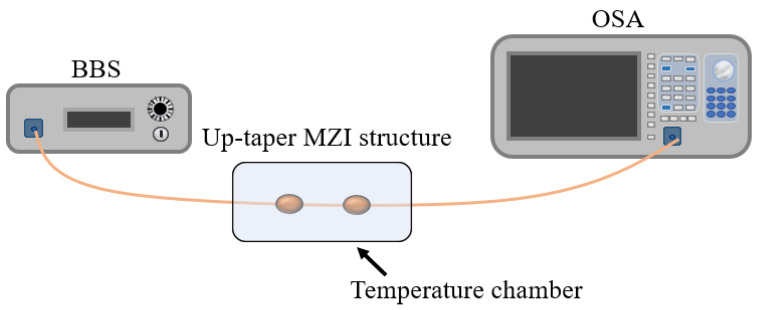
Experimental setup of Yb doped fiber up-tapered structure.

In order to verify whether the designed up-taper structure can produce the interference phenomenon, a BBS (Anyang SC-5) is used to connect the structure directly, and an optical spectrum analyzer (OSA, Yokogawa AQ6370D) is used to detect the wavelength movement, as shown in Figure 3.

**Figure 4:**
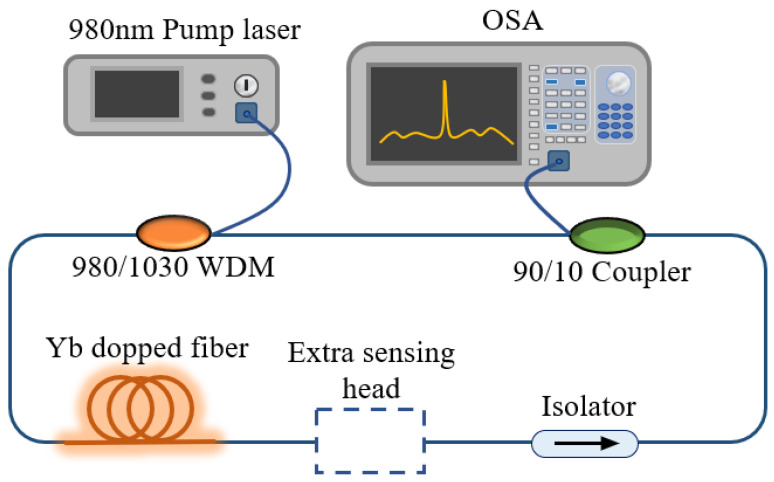
Traditional FRL sensing structure logic framework.

Furthermore, we used the FRL cavity for sensing experiments. Unlike traditional FRL systems, as shown in Figure 4, additional filters need to be designed for sensing monitoring. Yb doped fiber MZI can be used as filter, sensor and gain medium simultaneously. Without additional filtering structure, the system structure is simplified and the structure stability is increased. 

**Figure 5:**
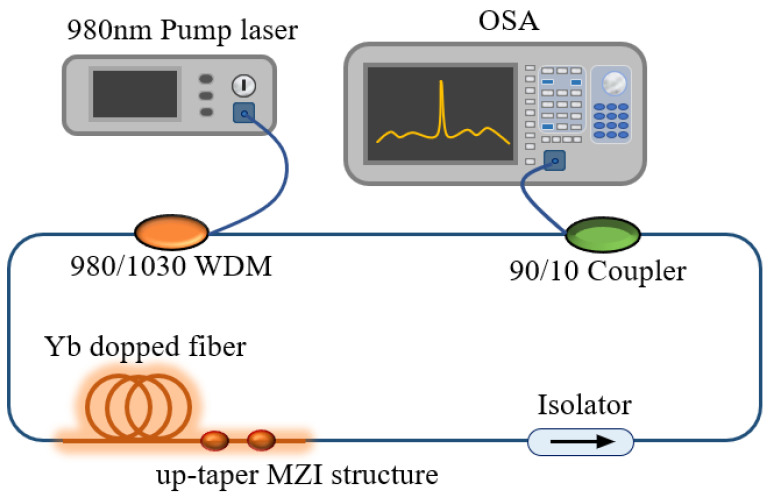
The intracavity sensing system of the FRL without extra sensing head.

The designed sensing structure is shown in Figure 5. The 980 nm pump source (PL-974-500-FC/APC-P-M) enters the ring cavity through the wavelength division multiplexer (WDM), gains the Yb doped fiber, and the tail end of the Yb doped fiber is designed into up-taper MZI structures through the fiber fusion splicer to produce interference effects. When the temperature changes, the corresponding interference spectrum will shift. An isolator is used to prevent optical reverse transmission from damaging devices. The modulated light passes through the 90:10 coupler and outputs 10% of the laser intensity to the OSA for monitoring. The remaining light intensity continues to circulate in the cavity. Based on this structure, we successfully monitored the temperature and RI characteristics of an Yb doped fiber MZI structure. 

## 3. Results

**Figure 6:**
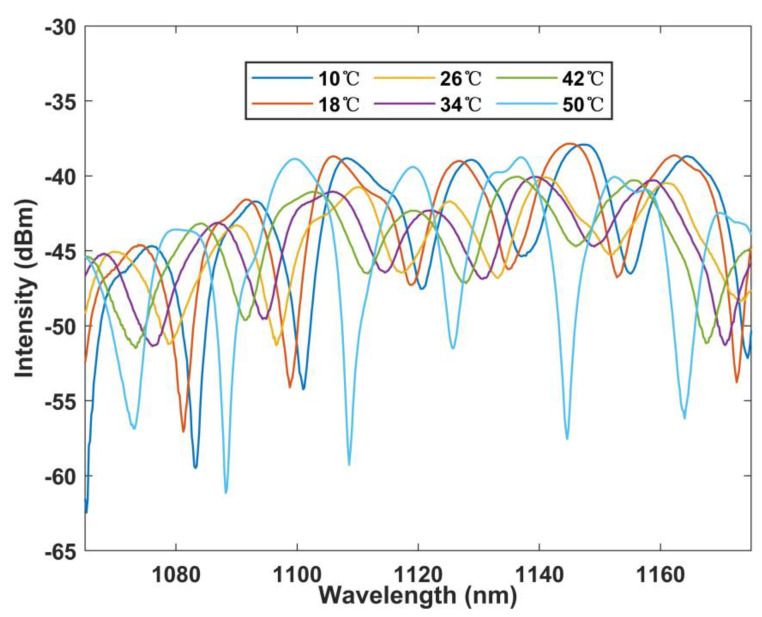
Wavelength versus temperature curve under BBS.

Figure 6 shows the rule of spectrum change with temperature under BBS as shown in the pre-experiment. In the range of 10–50 °C, the wavelength moves to short wavelength as the temperature rises.

**Figure 7:**
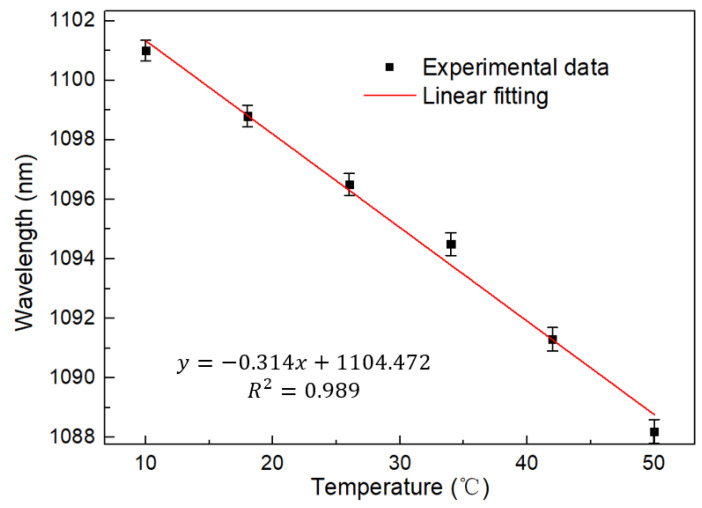
Linear regression curve of temperature with wavelength at BBS.

Figure 7 shows the linear fitting curve of wavelength with temperature when the measurement interval is 8 °C. The test sensitivity is as high as 0.314 nm/°C according to the figure. Meanwhile, the linear regression coefficient is 0.989. The linearity and feasibility of the sensor are proved. In addition, error bars are also provided, and it can be found that the error value of multiple measurements is less than 0.2 nm.

**Figure 8:**
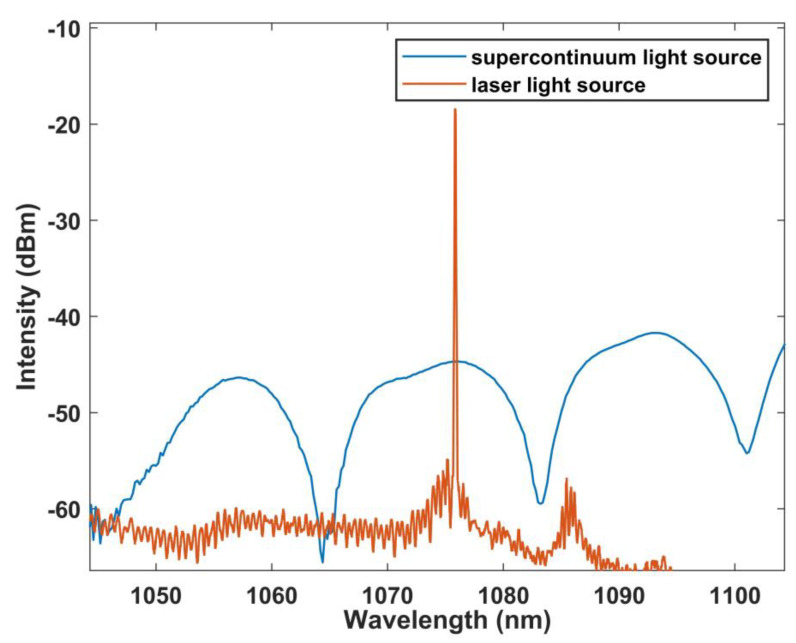
Corresponding curve of the output laser spectrum and interference spectrum.

Figure 8 shows the relationship between the output laser wavelength and the output result by using a BBS. It can be noted that the laser output is at the peak of the interference spectrum. This proves the accuracy of MZI working as a laser cavity filter. At the same time, the burr on the spectral edge may be caused by the unstable operation of the isolator.

**Figure 9:**
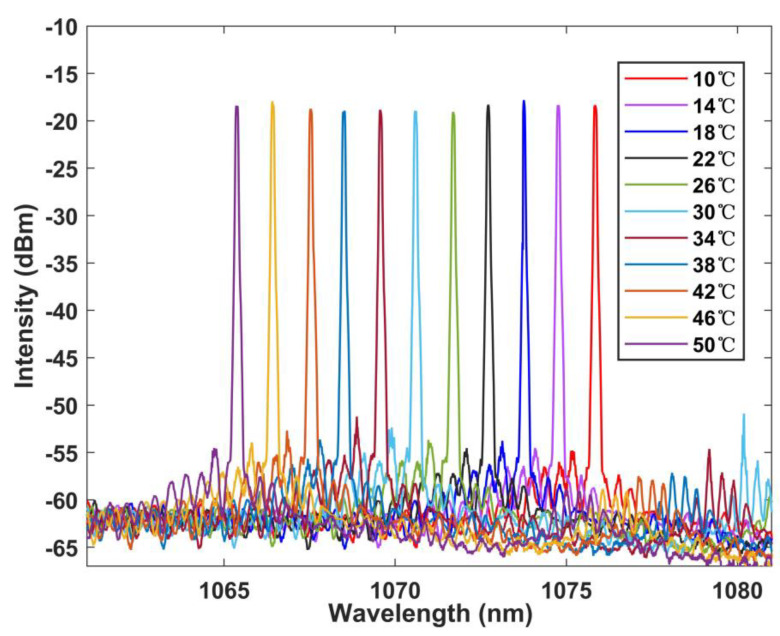
Wavelength versus temperature curve at FRL.

**Figure 10:**
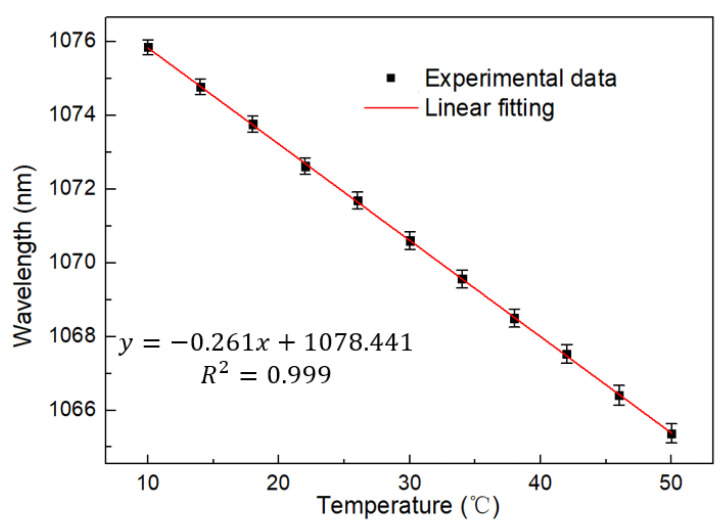
Linear regression curve of temperature with wavelength at FRL.

Figure 9 and Figure 10 show the relationship between the laser spectral shift and temperature in the FRL system. It can be found that the laser has a significant blue shift as the temperature increases. Good linearity can be obtained by testing every 4 °C in the range of 10–50 °C. The temperature sensitivity is 0.261 nm/°C, which is slightly lower than that of the BBS, possibly due to the deviation between the selected interference wave trough and the laser output. However, the laser still maintains good linearity with R squared up to 0.999 and error bar less than 0.15 nm, which proves the stability and consistency of the system.

**Figure 11:**
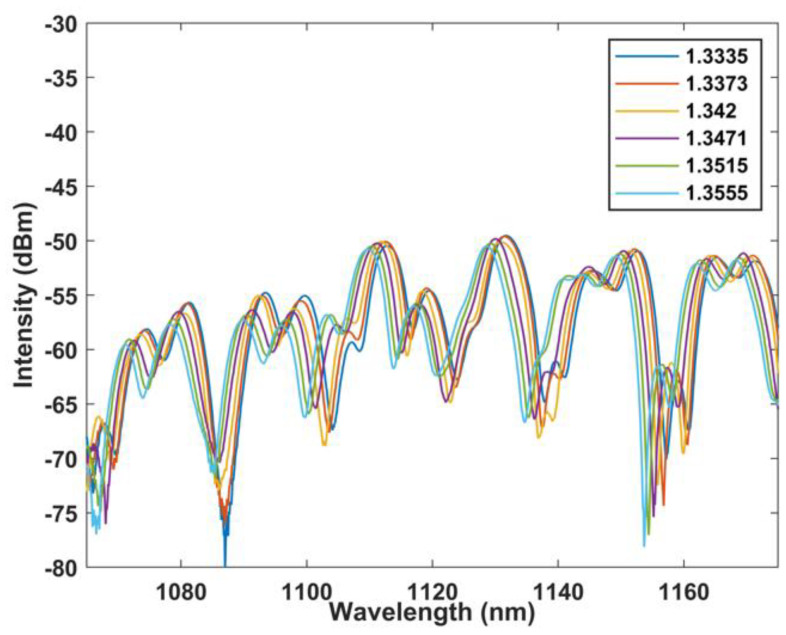
Wavelength versus RI curve at BBS.

**Figure 12:**
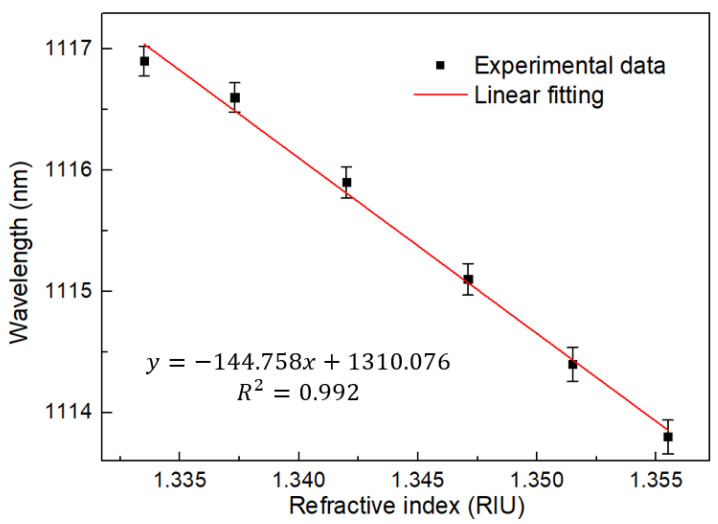
Linear regression curve of RI with wavelength at BBS.

In addition, the RI response characteristics of the designed MZI structure are analyzed. The results are shown in Figure 11 and Figure 12. Within the RI range of 1.3335–1.3555, the wavelength moves to shorter wavelength, and the corresponding response sensitivity is −144.758 nm/RIU, with a linear fit up to 0.992.

**Figure 13:**
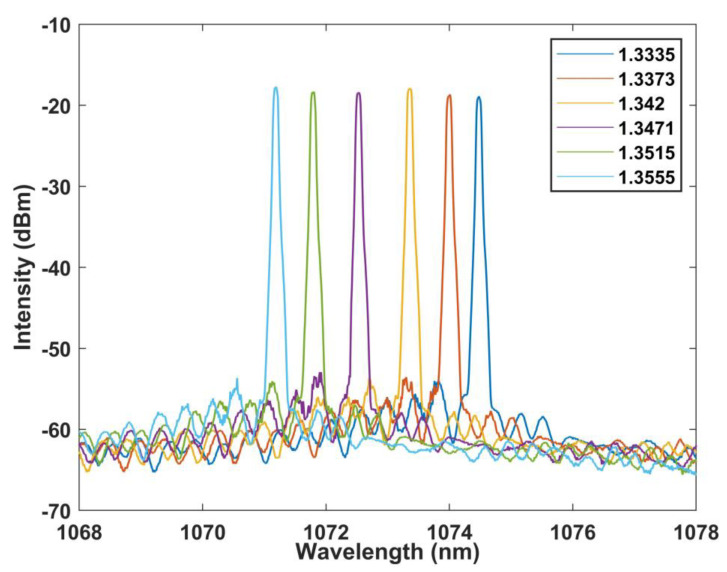
Wavelength versus RI curve at FRL.

**Figure 14:**
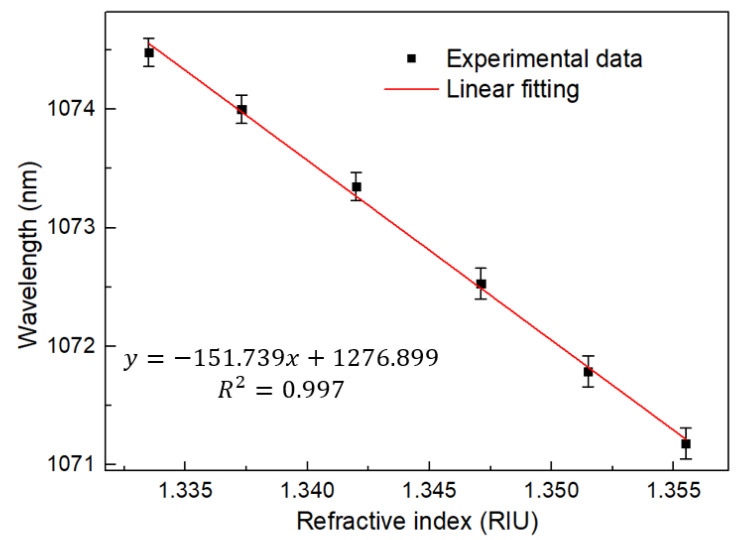
Linear regression curve of RI with wavelength at FRL.

The response characteristics of the laser light source are shown in Figure 13 and Figure 14, and its sensitivity is −151.739 nm/RIU with the linearity of 0.997. For the RI, we repeated the experiment for five times, and the error bar obtained is less than 0.13 nm. Therefore, it can be considered that the sensor has good repeatability. It must be discussed that the burr on the laser edge may be caused by the selected isolator being a polarization maintaining isolator.

**Figure 15:**
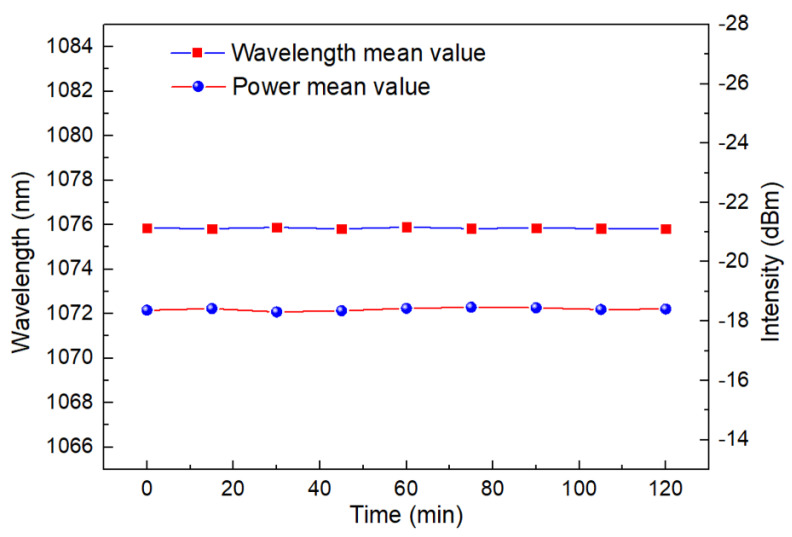
Laser wavelength and light intensity stability test at 10 °C.

Finally, we tested the laser stability at 10 °C for two hours. As shown in Figure 15, it can be concluded that the wavelength fluctuation is less than 0.2 nm. Light intensity fluctuation is less than 0.5 dB. This verifies the stability of the system. Although the sensor system needs further packaging design to become practical, the designed system based on FRL has an SNR higher than 40 dB and a linewidth less than 0.2 nm. This is incomparable to other BBS-based sensors.

## 4. Conclusions

In conclusion, we have designed an MZI based on the up-taper structure on Yb doped fiber in an FRL cavity. Different from traditional FRL sensors, in which additional filters are required as sensing units, the designed structure integrates the filter, sensor and gain medium into the laser cavity simultaneously. Thanks to the high thermal–optical coefficient of rare earth ions, the temperature sensitivity of the designed sensor is as high as 0.261 nm/°C. Furthermore, we tested the RI sensitivity and stability, and achieved 151 nm/RIU RI sensitivity. At the same time, the system maintains good stability within 2 h. Benefiting from the characteristics of the FRL itself, the designed structure has an SNR higher than 40 dB and a linewidth narrower than 0.2 nm. This provides an extremely powerful possibility for human life health detection and monitoring the normal operation of aircraft engines.

## Data Availability

Not applicable.
